# Supplementary figures and images for: Performance Characteristics of Next-Generation Sequencing for the Detection of Antimicrobial Resistance Determinants in Escherichia coli Genomes and Metagenomes
Source: mSystems. 2022 Jun 1;7(3):e00022-22. doi: 10.1128/msystems.00022-22 (PMC9238399; doi:10.1128/msystems.00022-22)

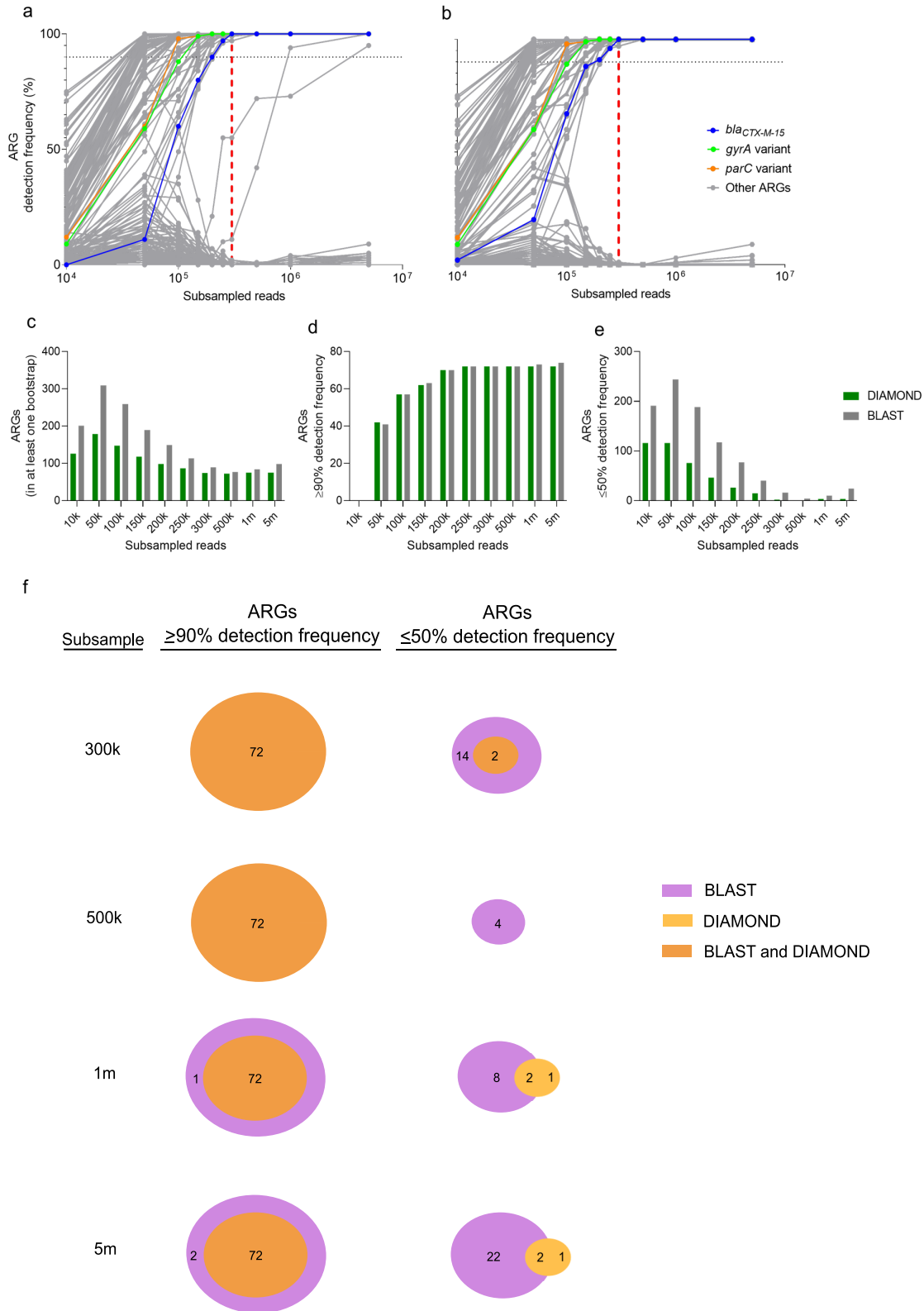

Supplement: FIG S1 [file msystems.00022-22-s0001.pdf]

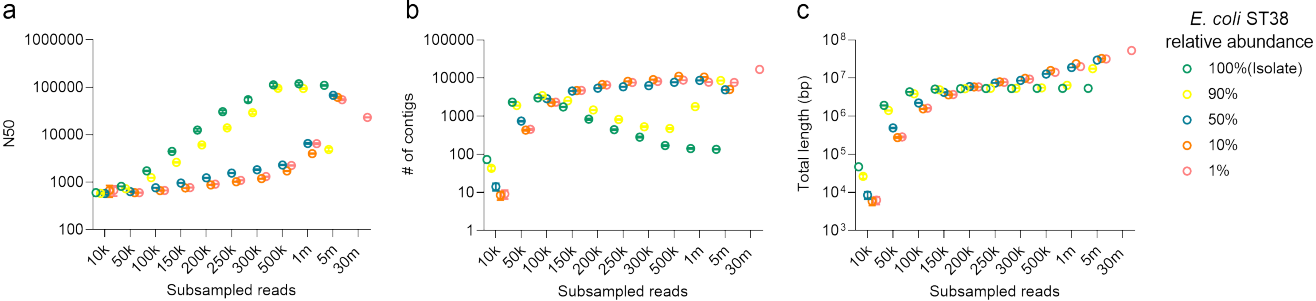

Supplement: FIG S3 [file msystems.00022-22-s0003.pdf]
