# Supplementary material for: Performance Characteristics of Next-Generation Sequencing for the Detection of Antimicrobial Resistance Determinants in Escherichia coli Genomes and Metagenomes
Source: mSystems. 2022 Jun 1;7(3):e00022-22. doi: 10.1128/msystems.00022-22 (PMC9238399; doi:10.1128/msystems.00022-22)
Supplement: FIG S2 [file msystems.00022-22-s0002.pdf]

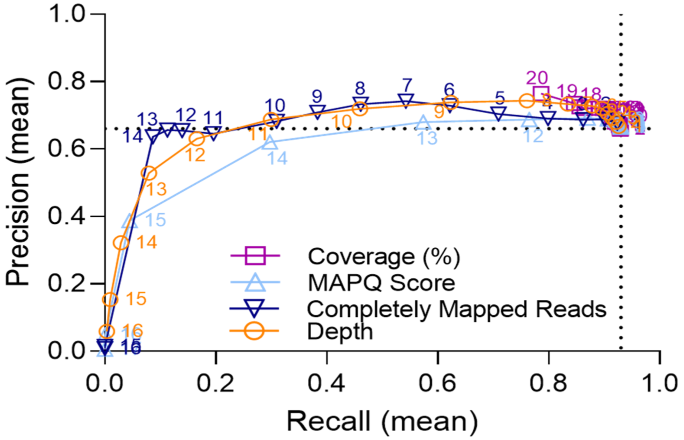

	Figure-associated cut-offs																			
**Filtration Method**	**1**	**2**	**3**	**4**	**5**	**6**	**7**	**8**	**9**	**10**	**11**	**12**	**13**	**14**	**15**	**16**	**17**	**18**	**19**	**20**
Coverage (%)	5	10	15	20	25	30	35	40	45	50	55	60	65	70	75	80	85	90	95	100
MAPQ Score	106	112	118	124	130	136	142	148	154	160	166	172	178	184	190	196	200			
Completely Mapped Reads	10	20	30	40	50	60	70	80	90	100	125	150	175	200	300	500				
Depth	0.5	1	2	3	4	5	6	7	8	9	10	11	12	13	14	15				
